# Emotional vulnerability and research capacity in medical postgraduates: toward understanding the reciprocal relationship and proposing targeted interventions

**DOI:** 10.3389/fpubh.2026.1813258

**Published:** 2026-09-29

**Authors:** Yanquan Liu, Xiaomei Hong, Jingdong Zhang, Honghua He, Xiaojun Chen, Jianzhen Shen

**Affiliations:** 1 Ganzhou People’s Hospital, The Affiliated Ganzhou Hospital, Jiangxi Medical College, Nanchang University, Ganzhou, Jiangxi, China; 2 Department of Public Health and Medical Technology, Xiamen Medical College, Xiamen, Fujian, China; 3 Department of Hematology, The Affiliated Hospital of Guangdong Medical University, Zhanjiang, Guangdong, China; 4 Department of Hematology, The Affiliated Hospital of Putian University, Putian, Fujian, China; 5 Department of Hematology, Fujian Medical University Union Hospital, Fuzhou, Fujian, China

**Keywords:** educational ecology, emotional vulnerability, intervention strategies, medical postgraduates, research capacity

## Abstract

As a backbone of medical research and clinical services, medical postgraduates require coordinated development of mental wellness and research capacity. In recent years, emotional vulnerability—characterized by poor emotion regulation, low stress resilience, and elevated risks of anxiety and depression—has become prevalent among this population and may impede the advancement of their research capacity. Drawing on resilience theory and educational ecology perspectives, this study develops a three-dimensional “psychological–cognitive–behavioral” analytical framework to examine theoretical associations between emotional vulnerability and diminished research capacity in medical postgraduates, alongside feasible intervention pathways. A two-dimensional “psychological – ecological” theoretical intervention framework is preliminarily constructed. This work offers an integrated interdisciplinary lens to inform research capacity cultivation for medical postgraduates and delivers actionable suggestions for reforms within medical graduate education systems.

## 1 Introduction

Medical postgraduate education, as a core component in cultivating high-level medical professionals, undertakes the crucial mission of advancing medical technological innovation and healthcare service development (1). However, alongside growing demands for medical research and intensified social competition, emotional health challenges have become increasingly prevalent among medical postgraduates (2, 3). Recent studies have documented a high prevalence of emotional disturbances, including anxiety and depression, within this population. It is hypothesized that emotional vulnerability may affect individual mental status and could potentially constrain research capacity via multiple intermediate pathways, such as elevated cognitive load, reduced motivation, and insufficient social support (4–6). Such observations point to potential structural shortcomings within conventional medical talent training frameworks concerning the coordinated relationship between psychological resilience cultivation and research capacity development.

Currently, academic research in this area is characterized by three main features (7–10): First, studies on psychological stress among medical postgraduates often focus on identifying stressors or implementing short-term interventions, lacking in-depth exploration of the dynamic interplay between emotional vulnerability and research capacity. Second, existing evaluation systems for research capacity tend to emphasize tangible outcomes, often neglecting the potential influence of emotional factors on research-related cognitive processes, including problem analysis, innovative thinking, and resilience to setbacks. Third, proposed countermeasures remain fragmented, failing to construct a systematic intervention framework that integrates educational model reform, support system enhancement, and coordinated policy guarantees. These limitations often leave medical universities struggling to effectively address the complex interplay of emotional and competency-related issues.

Consequently, this study adopts an interdisciplinary perspective integrating developmental psychology, educational ecology, and organizational behavior. Adopting a narrative synthesis approach grounded in existing empirical evidence and theoretical arguments, this paper systematically deconstructs the multidimensional correlational pathways between emotional vulnerability and weakened research capacity in medical postgraduates. Moving beyond traditional unidimensional analytical paradigms, this work establishes an explanatory model encompassing four levels: individual traits, supervisor interaction patterns, institutional support environment, and socio-cultural expectations. Based on this theoretical model, it further proposes targeted intervention strategies covering the entire “emotion-cognition-behavior” chain. This paper aims to provide a theoretical basis for the synergistic development of mental health promotion and research innovation capability enhancement among medical postgraduates, holding significant practical value for optimizing the cultivation system for top-notch medical talents in China.

## 2 Analysis of the current characteristics of emotion and research capacity among medical postgraduates

Emotional vulnerability among medical postgraduates is characterized by difficulties in emotion regulation and low stress tolerance. This study suggests that such emotional deficits potentially hinder their research-related cognitive processes. From the perspective of localized institutional context, existing research capacity evaluation systems place excessive emphasis on quantitative outputs yet overlook plausible influences of emotional factors on innovative thinking and setback resilience, with mental health indicators generally absent from these frameworks. Evidence from neurocognitive research demonstrates that chronic stress can impair executive function; findings from behavioral science further suggest a proposed feedback loop featuring “academic procrastination – self-denial”. Drawing on multidimensional existing evidence, this section seeks to lay a theoretical foundation for developing coordinated intervention systems.

### 2.1 Manifestations of emotional vulnerability and insufficient research capacity in medical postgraduates

The characteristic emotional vulnerability observed in medical postgraduates often manifests as a high propensity for anxiety, emotional exhaustion, and a diminished sense of self-efficacy. Under the dual demands of daily research and clinical responsibilities, their emotional problems frequently surface through somatic symptoms such as insomnia and headaches, as well as social withdrawal behaviors including avoidance of academic work and communication. Some medical postgraduates, potentially trapped in a state of “academic isolation” and lacking effective emotional support, may face an increased risk of depression (11). Concurrently, weakened research capacity primarily manifests as low efficiency in academic output, constrained innovative thinking, and inadequate problem-solving skills. Specific examples include repetitive experimental designs with low originality, logical confusion in academic writing, and reduced sensitivity to disciplinary frontiers. Some postgraduates, overly reliant on supervisory directives, may fall into a pattern of “instrumentalized research,” leading to delayed development of independent research skills and personal attributes such as emotional intelligence. Consequently, emotional vulnerability and weakened research capacity often exhibit “comorbid features.” For instance, individuals struggling with emotion regulation may develop “learned helplessness” in academia following experimental setbacks. Conversely, stagnation in research progress can trigger self-denial, creating a negative “emotion – cognition – behavior” feedback loop. It should be explicitly noted that this conceptual framework describing overlapping comorbid characteristics and reciprocal negative feedback constitutes a hypothesized model awaiting systematic empirical verification, rather than an empirically confirmed phenomenon.

### 2.2 Deficiencies and dilemmas in the research capacity evaluation system

1. *Evaluation bias driven by quantitative indicators*: Current research evaluation systems in the colleges and universities of some regions from China overemphasize explicit metrics such as publication count and impact factors, while neglecting the cultivation of implicit competencies including critical thinking, methodological rigor, innovativeness in the research process, teamwork, and academic resilience. This “outcome-oriented” assessment tends to foster short-term behaviors such as aestheticization of experimental data and low-quality submissions, thereby undermining the ethical foundation of scientific research.
2. *Systematic absence of mental health dimensions*: At present, evaluation frameworks in domestic medical universities generally fail to incorporate postgraduates’ mental health status into capacity assessment. This may lead to a disconnect between the emotional support needs of medical postgraduates and the requirements of research evaluation. Meanwhile, emotionally vulnerable postgraduates may experience reduced research efficiency due to psychological disturbances such as anxiety or depression, yet their struggles are often simplistically attributed to “insufficient capability,” further intensifying their psychological burden.
3. *Inadequate educational ecosystem alignment*: To date, significant homogenization of evaluation standards persists, failing to account for the dual tension between “clinical practice and basic research” across various medical disciplines. In practice, the dual burden of standardized residency training and fundamental research places medical postgraduates in a dilemma of time allocation. A unidimensional research assessment may exacerbate role conflict, creating a structural mismatch between capacity evaluation and actual needs.

### 2.3 Theoretical speculation of the negative emotion - research capacity cycle (based on existing empirical evidence)

This section summarizes consistent findings from published empirical studies to elaborate the potential association between negative emotion and research capacity decline, and further constructs a preliminary theoretical inference of a negative reciprocal cycle. Notably, the cycle model proposed below is a theoretical hypothesis that needs subsequent empirical verification among medical postgraduate populations.

1. *Evidence from neurocognitive mechanisms*: Functional neuroimaging study (12) indicates that chronic emotional stress can suppress prefrontal cortex activity, impairing executive functions such as planning and decision-making, which directly affects experimental design and data analysis efficiency. Concurrently, elevated levels of chronic stress hormones such as cortisol are associated with functional or structural impairments including reduced hippocampal volume, potentially compromising long-term memory and knowledge integration (13). Based on these neurocognitive empirical findings, we theoretically infer that persistent negative emotions may undermine the cognitive foundation of research capacity development.
2. *Longitudinal findings from behavioral science*: Longitudinal studies (14, 15) reveal that emotionally vulnerable individuals often exhibit a “high motivation - low efficacy” pattern in the early stages of research, with cognitive resources frequently consumed by emotional rumination, leading to fragmented attention. Over time, this population is more prone to behavioral patterns characterized by “academic procrastination - intensified guilt - further declines in efficiency”. Synthesizing these behavioral empirical results, we propose a theoretical self-reinforcing feedback loop hypothesis between negative emotion and impaired research performance.
3. *Moderating effects of the educational ecosystem*: It is undeniable that supervisor support and team inclusivity are critical variables in breaking the negative cycle. For example, supervisors with high mentoring efficacy can help postgraduates rebuild academic confidence through goal decomposition strategies and growth-oriented feedback. Meanwhile, collaborative laboratory cultures can mitigate the direct impact of emotional vulnerability on research capacity via resource sharing and stress buffering. These educational ecological findings provide an empirical basis for our theoretical framework of situational moderating mechanisms.

In summary, Figure 1 illustrates the mechanistic framework underlying the current characteristics of emotion and research capacity among medical postgraduates. Indeed, the epidemiological characteristics of emotion and research capacity among medical postgraduates fundamentally represent the co-evolutionary outcome of an imbalance between individual psychological traits and the educational ecosystem. Addressing this dilemma requires a tripartite approach: accurately identifying vulnerable populations, restructuring evaluation paradigms, and optimizing ecological support. This shift entails transforming medical education from a “selection-and-elimination” model to a “resilience cultivation” paradigm. Future research should further integrate neuroeducation and complex systems theory to establish dynamic monitoring and early intervention systems.

**Figure 1:**
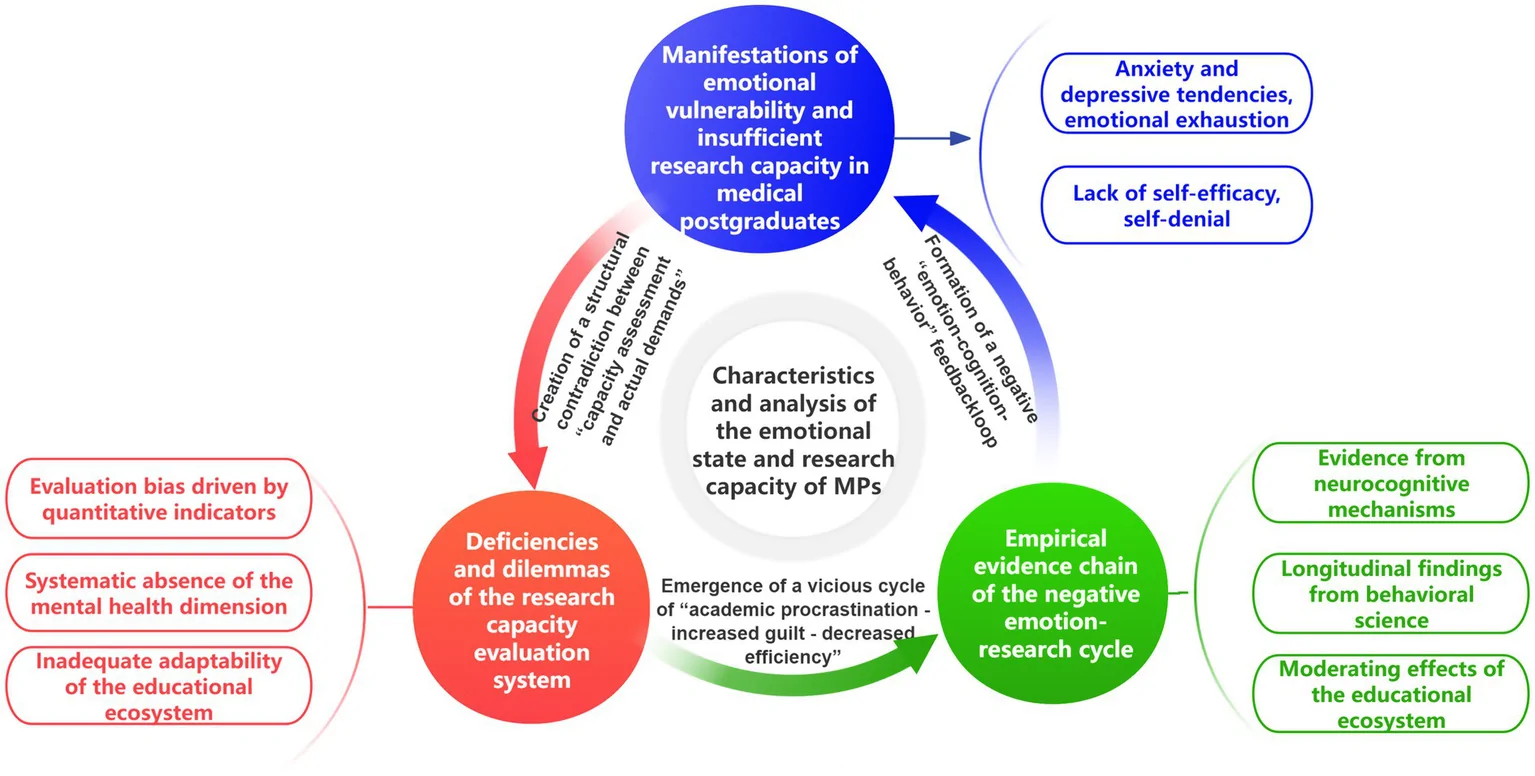
Mechanistic framework of the current characteristics of emotion and research capacity in medical postgraduates.

## 3 A multidisciplinary theoretical mechanistic framework for emotional vulnerability and insufficient research capacity in medical postgraduates

The issues of emotional vulnerability and weakened research capacity among medical postgraduates are fundamentally affected by the interplay of multiple dimensions, including psychological, educational, and physiological factors. Drawing on published empirical evidence and multidisciplinary theories, this section systematically constructs a preliminary theoretical mechanistic framework from the tripartite perspectives of psychology, pedagogy, and physiology/biology, aiming to provide theoretical support for optimizing the medical postgraduate training system. It is worth emphasizing that this multi-factor interactive mechanism is a theoretical proposition proposed in this Perspective, which remains to be quantitatively verified by subsequent empirical research.

### 3.1 Psychological perspective: inhibitory effect of chronic stress on cognitive function

1. *Chronic stress and cognitive resource depletion*: As previously noted, medical postgraduates are chronically exposed to the dual burdens of intensive research tasks and high-pressure clinical practice. This chronic stress state can persistently activate the hypothalamic–pituitary–adrenal (HPA) axis, leading to abnormally elevated cortisol levels (16). Furthermore, research (17, 18) indicates that excessively high cortisol levels can suppress prefrontal cortex function, thereby impairing executive control abilities such as attention allocation and planning, which subsequently affects the efficiency of medical postgraduates in research analysis and experimental processes. Concurrently, chronic stress triggers emotional rumination, consuming substantial cognitive resources and manifesting as “fragmented attention” and “decisional procrastination,” which exacerbates low research efficiency.
2. *Emotion regulation failure and attenuation of academic motivation*: Emotionally vulnerable individuals are often more prone to falling into a “negative affect – cognitive rigidity” cycle. For instance, if anxiety triggered by routine experimental failures is not promptly regulated, it can induce “learned helplessness” in academia, characterized by a persistent tendency to avoid research goals. Neuroscientific evidence (19) demonstrates that chronic emotional stress can reduce hippocampal neuroplasticity, impairing knowledge integration and long-term memory capabilities, thereby further constraining innovative research thinking.
3. *The buffering effect of psychological resilience*: Psychological resilience, as a core protective factor, can effectively mitigate the inhibitory effects of stress on cognitive function through emotion regulation strategies such as cognitive reappraisal and problem-focused coping, as well as external support networks including supervisor guidance and peer assistance. Individuals with high resilience are more adept at transforming pressure into challenging motivation, thereby sustaining research resilience.

### 3.2 Pedagogical perspective: interaction between supervisory styles and self-efficacy

1. *Potential risks of authoritarian supervision*: In practice, some supervisors in China adopt a “results-oriented” management style. As an illustrative anecdotal example, first-year graduate students may be required to publish Chinese core journal articles or even high-level SCI papers within a short timeframe, a requirement that is highly likely to trigger goal anxiety and self-doubt. Supervisory patterns overemphasizing paper output and short-term achievements while neglecting systematic cultivation of research competencies can easily diminish student self-efficacy, encourage engagement in “instrumentalized research,” and inhibit students’ abilities for autonomous academic exploration.
2. *Empowering mechanisms of growth-oriented supervision*: Supervisors who employ a dual-path “support-feedback” approach – through goal decomposition, recognition of incremental achievements, and growth-oriented feedback emphasizing effort rather than innate talent – can effectively enhance the research self-efficacy of medical postgraduates. Studies (20, 21) indicate that positive, proximal communication from supervisors, coupled with the provision of necessary academic guidance and resource support, can strengthen students’ sense of control over research challenges and facilitate the formation of intrinsic motivation.
3. *Systematic alignment of the educational ecosystem*: Research capacity cultivation should be embedded within a synergistic “individual – supervisor – institution” ecosystem. For instance, dynamic academic resource platforms, such as shared databases and interdisciplinary collaboration mechanisms, can lower the barriers to research entry. Meanwhile, a diversified evaluation system that balances process quality and innovativeness can alleviate the anxiety associated with a “publication-only” focus, thereby reconstructing students’ perceptions of sound research values.

### 3.3 Physiological and biological foundations: intrinsic individual functions and effect correlations

1. *HPA axis activation and hippocampal atrophy*: Evidence concerning chronic stress responses is primarily derived from studies of PTSD veterans and chronically stressed adult populations (22). These studies show that sustained elevation of cortisol under prolonged stress can lead to abnormal activation of glucocorticoid receptor signaling pathways. Such pathway disturbances are associated with suppressed hippocampal neurogenesis and reduced hippocampal volume within these specific cohorts. Given the central role of the hippocampus in memory consolidation and episodic learning, we hypothesize that analogous hippocampal dysfunction might impair medical postgraduates’ ability to conduct literature synthesis and hinder interdisciplinary knowledge transfer (23). We note that this hypothesized extension from veteran and general adult samples to medical postgraduates is not directly validated in medical trainee populations. Findings from preclinical animal models offer mechanistic background supporting this stress-related neural injury cascade: rodents subjected to chronic stress display diminished dendritic complexity of hippocampal neurons alongside impaired spatial memory (24). These animal data cannot be directly translated to human medical postgraduates. Nonetheless, translating these preclinical and veteran-population observations to medical postgraduates represents a substantial inferential leap, and the proposed cognitive consequences in this specific student group should be regarded as tentative rather than definitive.
2. *Epigenetic regulation and potential for reversible interventions*: Current empirical studies demonstrating cortisol-mediated epigenetic modifications (e.g., DNA methylation) of brain-derived neurotrophic factor and subsequent regulation of brain synaptic plasticity are based on general stressed adult populations (25). Clinical intervention trials targeting stress-affected adult groups (26) have verified that mindfulness-based stress reduction training can effectively reduce peripheral cortisol levels and is accompanied by increased hippocampal gray matter density. By extrapolation, it could be speculated that chronic stress-induced cognitive impairment in medical postgraduates may also have reversible characteristics through similar intervention strategies. Importantly, these intervention effects have not been tested specifically among medical postgraduates. Nevertheless, it should be emphasized that the reversibility and targeted intervention mechanisms for medical postgraduates’ stress-related cognitive deficits still lack population-specific empirical validation, and rigorous scientific verification of effective intervention paradigms for this group remains to be supplemented.
3. *Biomarker-guided early warning*: We proposed an original intervention strategy that interrupting the bidirectional feedback between emotional vulnerability and diminished research capacity in medical postgraduates may rely on building an integrated “psychological - educational - physiological” intervention framework. Firstly, on the psychological level, stratified psychological resilience training (e.g., cognitive behavioral therapy, stress inoculation training) and dynamic mental health monitoring systems have been validated in general stressed adult populations. The efficacy of these approaches for medical postgraduates remains to be confirmed. These tools are expected to relieve emotional vulnerability amid academic pressure for medical postgraduates. Secondly, on the educational level, optimising supervisor training to cultivate growth-oriented mentoring competency and establishing a balanced training model coordinating clinical practice and research requirements may reduce external stressors for medical postgraduates. Lastly, on the individual physiological level, biofeedback-assisted stress management combined with neuroplasticity training is presumed to expand cognitive reserve and counteract stress-associated neural and cognitive impairments. This presumption draws on evidence from general adult and clinical samples and requires dedicated testing within medical postgraduate cohorts.

In summary, the multidisciplinary mechanistic diagram illustrating the hypothesized interplay between emotional vulnerability and insufficient research capability among medical postgraduates is presented in Figure 2. Future research in health science education and medical education should further integrate neuroeducation and complex systems theory to explore precise, multidisciplinary intervention pathways, thereby facilitating a paradigm shift from a “screening-and-elimination” model to a “resilience-cultivation” approach in the training of medical postgraduates.

**Figure 2:**
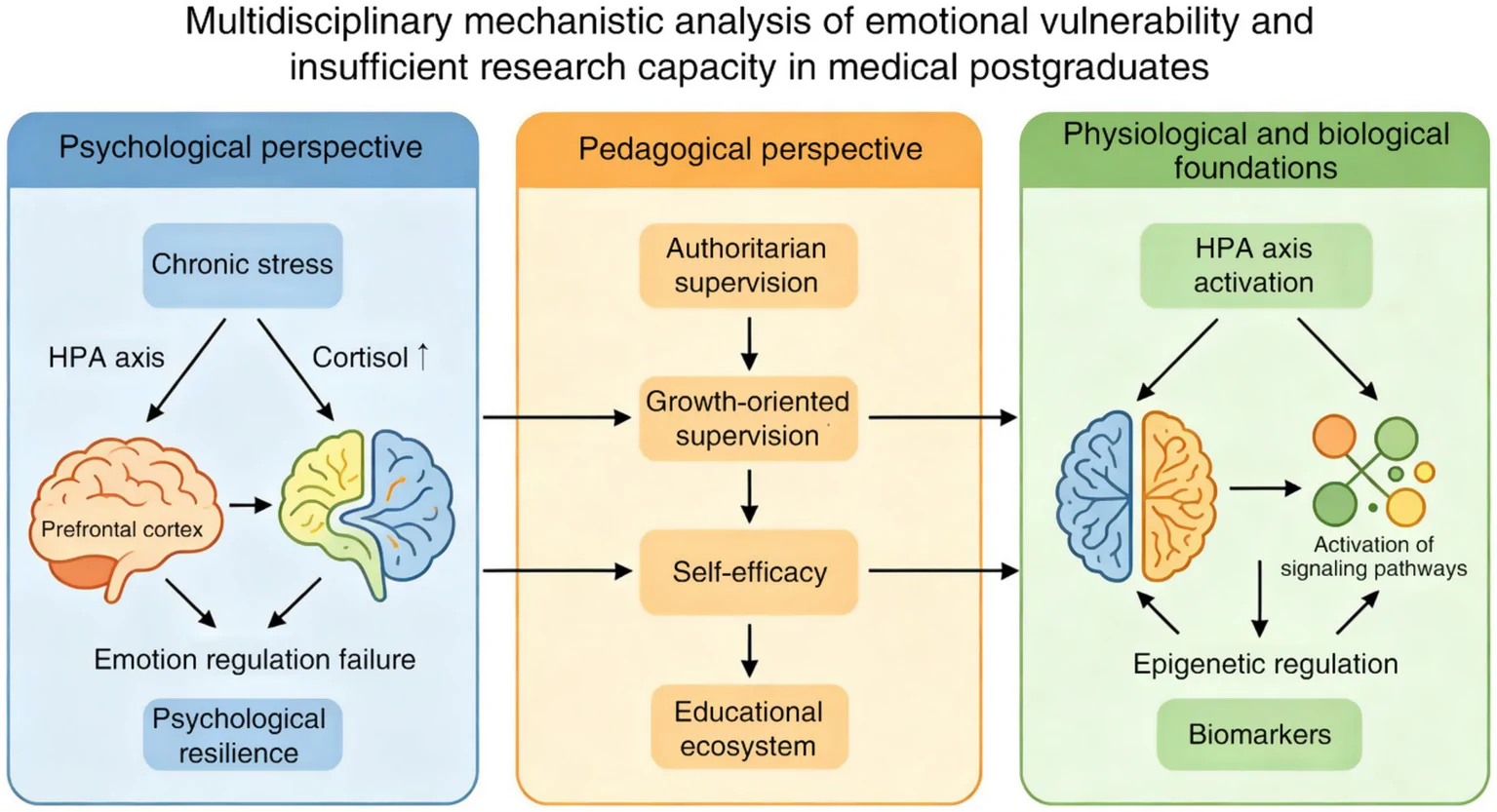
Mechanistic diagram of the multidisciplinary mechanisms underlying emotional vulnerability and insufficient research capability in medical postgraduates.

## 4 Influencing factors of emotional vulnerability and insufficient research capacity in medical postgraduates

Targeting Chinese medical postgraduates, this section systematically explores the dual predicament of emotional vulnerability and inadequate research capacity during their academic and professional development. The formation of this dual dilemma stems from the interactive coupling of individual psychological traits, localized educational institutional mechanisms, and unique sociocultural contexts in China’s medical education ecosystem. Different from Western medical graduate training systems, China’s medical postgraduate cultivation is embedded in distinctive supervisor mentorship modes, university research evaluation systems, and culturally specific professional narratives, which jointly shape the developmental status of medical trainees (27, 28). This section deconstructs the multi-dimensional influencing mechanisms of the above problems based on China’s localized educational policies and cultural backgrounds, aiming to enhance the contextual adaptability and pedagogical validity of the research findings.

### 4.1 Individual factors: personality traits and the self-discipline of coping strategies

As the core psychological foundation regulating individual academic behaviors, personality traits determine the cognitive perception, emotional response, and behavioral decision-making of Chinese medical postgraduates when facing research pressure (29). Neurotic individuals are more prone to negative cognitive rumination dominated by anxiety and depression in academic scenarios. Faced with experimental failures, manuscript rejection, or conflicts between clinical rotation tasks and scientific research, they tend to attribute setbacks to personal incompetence rather than objective institutional or environmental factors. This negative attribution bias triggers a self-reinforcing cycle of academic self-denial, which gradually weakens academic concentration and reduces research efficiency, further exacerbating emotional vulnerability.

In contrast, medical postgraduates with low conscientiousness lack systematic and goal-oriented research planning awareness. Restricted by the dual pressure of clinical internship and academic research unique to China’s medical training system, they are easily overwhelmed by fragmented tasks and tight time schedules, resulting in delayed research progress and accumulated academic pressure (30). The heterogeneity of coping strategies further differentiates individual developmental outcomes. Influenced by the traditional educational concept of “excellence in all aspects” in China, many medical postgraduates hold rigid perfectionist expectations for their academic and clinical performance. Their excessive adoption of emotion-focused coping strategies consumes massive psychological resources, while failing to establish standardized knowledge management and research progress mechanisms. Additionally, some trainees adopt avoidant coping characterized by cognitive inertia: they escape persistent research pressure through social media entertainment and passive procrastination, which temporarily relieves anxiety but continuously erodes academic self-efficacy, forming a vicious cycle of insufficient research capacity and negative emotion accumulation.

### 4.2 Educational-ecological factors: institutional pressure and the paradox of resource allocation under China’s medical education reform

China’s university research evaluation system and graduate supervisor mentorship system are the core institutional factors inducing emotional vulnerability and research capacity deficiencies among medical postgraduates, and their institutional reform evolution directly shapes the academic growth environment of medical trainees (28). For a long time, China’s medical graduate education has been constrained by the quantitative-oriented academic evaluation mechanism centered on SCI paper output. This single-dimensional assessment system has constructed a distorted academic value orientation in domestic medical colleges and universities, forcing medical postgraduates to pursue “quick academic achievements” to meet graduation and assessment requirements, even lead to academic misconduct (31). Such homogenized institutional assessment pressure not only restricts the innovative research thinking and personalized research interest development of medical postgraduates but also induces persistent institutional anxiety, significantly increasing their emotional vulnerability risk (32).

Beyond the evaluation system, the localized supervisor mentorship system in China constitutes another key restrictive factor for medical postgraduates’ research growth. Different from the decentralized tutoring mode in Western countries, China’s medical graduate training adopts a typical supervisor-responsible system, where supervisors serve as the primary persons in charge of students’ academic cultivation and career guidance (28, 33). In the context of medical education reform, although domestic universities in China have gradually promoted multi-mentor collaborative training mechanisms and standardized supervisor performance management, many medical supervisors still suffer from insufficient guidance energy, single tutoring methods, and disjointed clinical-research guidance (34). A large number of medical postgraduates are in a state of “passive academic grazing” without systematic research thinking training, standardized experimental guidance, and targeted academic planning. The lack of continuous and effective supervisor support deprives students of behavioral guidance and psychological security in the research process, simultaneously hindering the accumulation of research capabilities and aggravating negative emotional experiences (35).

In terms of educational resource allocation, the disciplinary isolation of domestic medical colleges and the scarcity of interdisciplinary collaboration platforms further restrict the research development of medical trainees. Most clinical postgraduates are confined to single clinical departments, lacking effective channels to transform clinical practical problems into standardized scientific research questions, resulting in difficulties in research design, innovation, and implementation (36). In recent years, China’s higher medical education has launched a series of institutional reforms, including diversified academic evaluation reform, supervisor team construction, and clinical-research integrated training system optimization. However, the reform implementation is still in the transitional stage, and the institutional paradox of rigid assessment and insufficient high-quality educational resources has not been completely resolved, which continues to restrict the high-quality cultivation of medical postgraduates’ research capabilities and emotional health (37).

### 4.3 Sociocultural factors: the cognitive coercion of the professional sanctity narrative

The emotional vulnerability and research capacity dilemmas of Chinese medical postgraduates are deeply rooted in the unique sociocultural narrative of China’s medical industry, especially the culturally embedded “angel in white” professional sanctity discourse (38). Different from the neutral professional positioning of medical staff in Western cultural contexts, Chinese society has long endowed medical practitioners with sacred moral attributes and altruistic professional expectations, forming a fixed cultural narrative of medical professionalism centered on dedication, perseverance, and omnipotence (39). This glorified professional narrative constructs a rigid idealized self-cognition for medical postgraduates at the initial stage of their career development. Most trainees internalize the social expectation of “professional sanctity” and form the cognitive stereotype that “medical workers must be excellent and unyielding”. They often ignore their own emotional needs and psychological pressure in the process of research exploration and clinical training, and are unwilling to admit academic setbacks and ability deficiencies, which greatly intensifies emotional vulnerability (40, 41). The sharp contradiction between the perfect professional identity shaped by the “angel in white” narrative and the real experience of frequent research failures, manuscript rejections, and research bottlenecks in academic practice creates a serious cognitive fissure in the professional identity construction of medical postgraduates.

Furthermore, influenced by the traditional Chinese cultural concept of “diligence leads to success”, medical postgraduates often attribute research plateaus and academic difficulties entirely to their own insufficient efforts. This cultural cognitive coercion triggers severe self-blame and self-doubt, exacerbating emotional anxiety and burnout (42, 43). For clinical medical postgraduates who excel in clinical operational skills, the inability to transform clinical advantages into academic research achievements easily leads to “academic self” identity dissociation, forming a role conflict between “clinical practitioner” and “research explorer.” This long-term role confusion and cognitive dissonance eventually lead to the loss of academic focus, insufficient research motivation, and continuous deterioration of emotional states. Collectively, the localized sociocultural narrative, coupled with China’s unique medical education institutional environment, jointly constitutes the external situational constraints for the dual predicament of Chinese medical postgraduates. Optimizing the professional cultural narrative, improving the localized supervisor guidance system and diversified research evaluation mechanism, and building a sound emotional support system are crucial ways to alleviate emotional vulnerability and improve the research comprehensive quality of domestic medical postgraduates.

In summary, the mechanistic diagram of the influencing factors underlying emotional vulnerability and insufficient research capability among medical postgraduates is presented in Figure 3.

**Figure 3:**
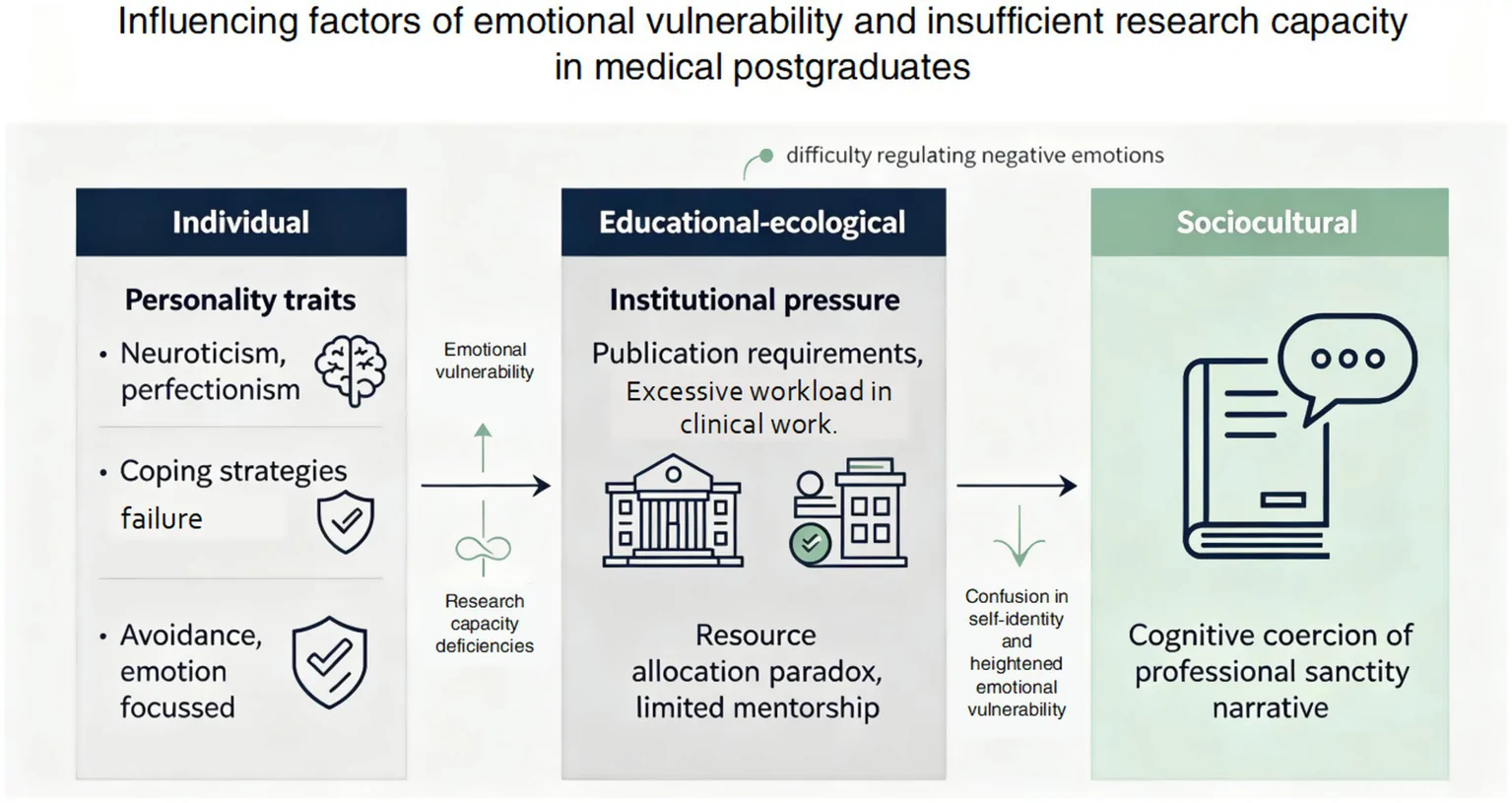
Mechanistic diagram of the influencing factors of emotional vulnerability and insufficient research capability in medical postgraduates.

## 5 A multi-dimensional proposed framework of potential intervention strategies for emotional vulnerability and insufficient research capacity in medical postgraduates

The developmental predicament faced by medical postgraduates is, in essence, a microcosm of the conflict between instrumental rationality and subjective needs within the context of modernity. Traditional fragmented intervention models are inadequate for addressing this systemic crisis. Against this theoretical backdrop, this study proposes a tentative multi-dimensional intervention framework that integrates individual cognitive restructuring, transformation of supervisory paradigms, and the reconstruction of the institutional support ecosystem. This framework is conceptualized to facilitate a potential paradigm shift from remedial repair to developmental cultivation for medical postgraduate training, while its practical effectiveness remains to be empirically verified.

### 5.1 Individual level: proposed application of mindfulness-based cognitive therapy (MBCT)

Integrating mindfulness meditation and cognitive restructuring techniques, MBCT is theoretically applicable to the novel hypothesized self-reinforcing feedback process proposed in this study, which is characterized by “cognitive rigidity – emotional fixation” among medical postgraduates. Its potential supportive effects are inferred from existing theoretical evidence and can be preliminarily interpreted across three cognitive dimensions. First, attentional regulation: MBCT may train postgraduates to decouple automatic negative responses to research setbacks, which is hypothesized to interrupt the vicious cognitive loop of “experimental failure → ability attribution → self-denial”. Second, cognitive defusion: this approach may guide individuals to reframe rigid self-narratives such as “I am not suited for research” toward objective observation of “I am currently experiencing research challenges”. Such cognitive reframing has the theoretical potential to mitigate academic identity crises and strengthen cognitive resilience against adverse events including repeated experimental failures and manuscript rejection. Third, value re-anchoring: values clarification exercises may help postgraduates distinguish extrinsically driven from intrinsically oriented research motivations, which plausibly restores academic curiosity and research enthusiasm and alleviates negative emotions. As a theoretically grounded strategy facilitating concurrent cognitive and neural adjustment, MBCT practice provides a plausible preliminary pathway to mitigate psychological barriers that may restrict the development of research capacity, though targeted empirical validation among medical postgraduate populations is still lacking (44, 45).

### 5.2 Supervisor level: proposed paradigm shift toward a “growth mindset” orientation

The role reconstruction of supervisors in medical postgraduate training necessitates a tentative paradigm shift from “output-oriented supervisor” to “growth mindset cultivator”, as a theoretically informed optimization direction for supervisory practice. The proposed optimization strategies are outlined as follows. First, restructuring the feedback discourse system: abandoning evaluative language rooted in a fixed “gift mindset”, such as “lacking research talent”, in favor of process-oriented evaluations. Emphasizing the scientific principles of neuroplasticity and deliberate practice may help dispel students’ misconceptions about fixed academic ability, with tentative benefits for reducing research anxiety. Second, cultivating potential academic resilience mechanisms: constructing hypothetical “controlled failure” training scenarios, such as deliberately incorporating methodological pitfalls in project design, to guide students in experiencing a positive “setback–growth” cycle through error correction. This conceptual practice aligns with self-efficacy theory and is speculated to enhance research confidence through the accumulation of mastery experiences, without conclusive population-specific evidence. Third, reactivating the modeling effect: supervisors’ conscious disclosure of their own research setback experiences may deconstruct the stereotyped image of the “perfect successful scholar”, thereby potentially lowering students’ achievement anxiety threshold.

Notably, this study emphasizes precautionary considerations for practical implementation. It is necessary to guard against the inertial power dynamics inherent in traditional apprenticeship models, preventing the growth mindset cultivation model from devolving into a new disciplinary tool. Genuine paradigm transformation theoretically requires supervisors to relinquish the identity of “absolute authority” and foster a dialogic academic atmosphere; however, the practical operability and long-term effects of this optimization approach require further empirical exploration.

### 5.3 Institutional level: proposed construction of a dual-support platform for research development and psychological nourishment

The structural deficiencies of existing institutional support systems for medical postgraduate training are theoretically identified as key contributors to students’ emotional vulnerability and insufficient research capacity. To address these systemic limitations, this study puts forward prospective, theoretically grounded institutional optimization strategies to construct a symbiotic ecosystem of “research capacity development and psychological nourishment”. All proposed measures are conceptual improvements rather than empirically validated interventions, with specific recommendations as follows:

1. *Interdisciplinary supervisory panels*: We propose forming joint guidance teams comprising clinical experts, basic science researchers, and psychological consultants. Theoretically, incorporating cognitive psychology perspectives during the project design phase may simultaneously improve postgraduates’ research question focusing ability and ambiguity tolerance, thereby facilitating the synergistic development of research thinking and psychological qualities. The practical efficacy of this interdisciplinary supervision model remains to be verified by follow-up empirical studies.
2. *Embedded psychological workshops*: We propose integrating mindfulness training, cognitive restructuring and other psychological adjustment techniques into routine research skills training courses. For instance, embedding attention training into literature review sessions and emotion management strategies into thesis writing guidance may theoretically break the dualistic “research versus psychology” separation in traditional knowledge transmission.
3. *Academic psychological counseling services*: We propose establishing a new role of “academic growth advisors”, preferably filled by experienced academic researchers. Distinct from the pathologizing orientation of traditional psychological counseling, these advisors, leveraging their disciplinary research experience, may accurately identify domain-specific psychological distress (e.g., existential anxiety induced by repeated experimental failures) and provide targeted academic-psychological support. This service model is a tentative innovative proposal and lacks population-based practical validation.
4. *Flexible evaluation system optimization*: We propose allowing postgraduates to apply for temporary “research adjustment pauses” for psychological regulation and incorporating psychological growth indicators into the academic capability assessment framework. This institutional optimization idea may theoretically realize coordinated development at the micro individual and meso institutional levels, while its practical rationality and implementation effect need further systematic evaluation.

In summary, the mechanistic framework for the multi-dimensional construction of intervention strategies for emotional vulnerability and insufficient research capacity in medical postgraduates is illustrated in Figure 4. The intervention measures across the aforementioned levels are not merely additive but generate synergistic effects through the interaction of “cognitive reshaping – relationship reconstruction – institutional innovation”. The ultimate goal is not to eliminate emotional vulnerability but to transform it into a psychological resource for academic innovation; nor is it simply to enhance research output, but to rebuild the intrinsic value of medical research. Only through this approach can medical postgraduates truly grow into integrated subjects embodying both scientific rationality and humanistic concern, thereby achieving the unity of professional value and life meaning while addressing complex medical challenges.

**Figure 4:**
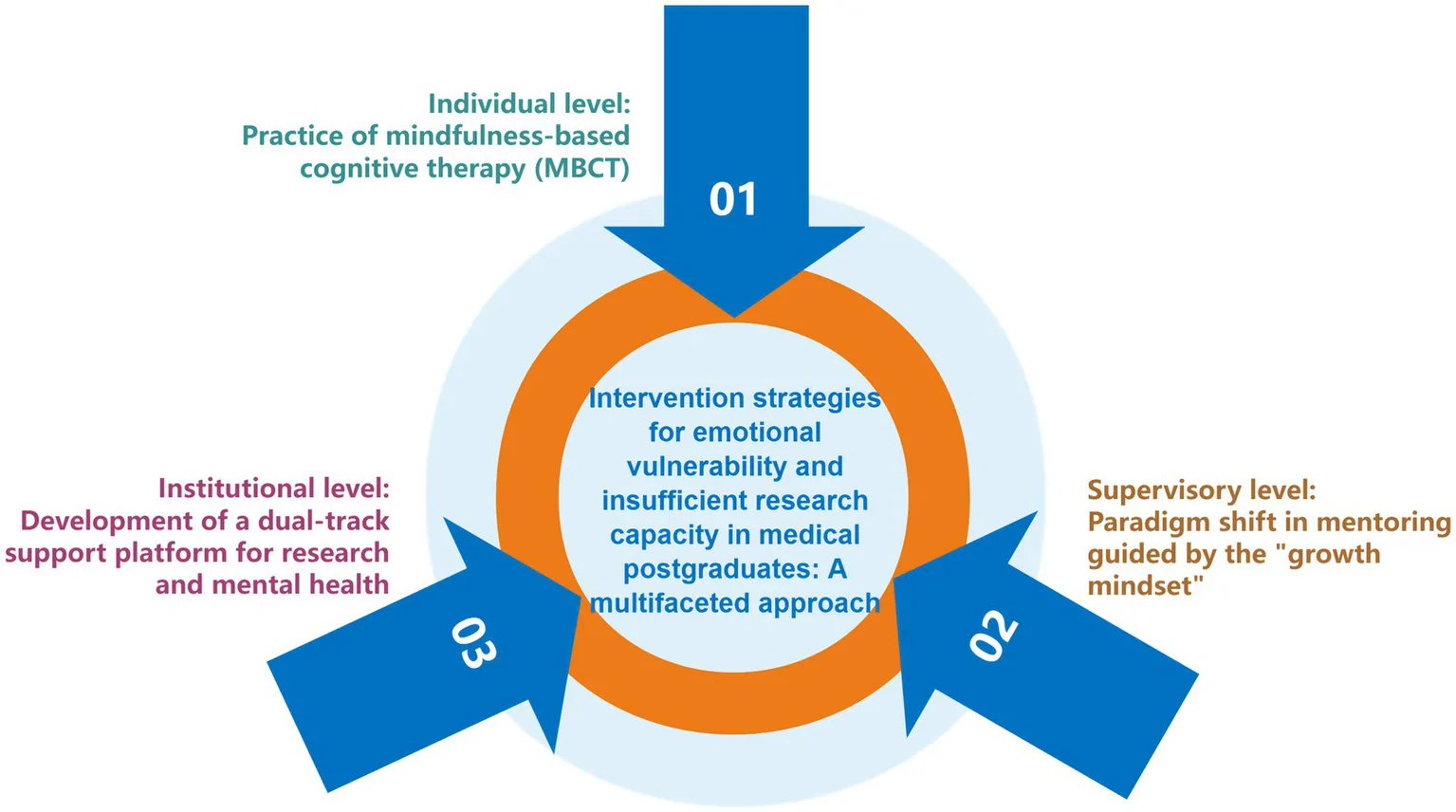
Conceptual mechanistic framework of proposed multi-dimensional intervention strategies for emotional vulnerability and insufficient research capacity in medical postgraduates.

## 6 Future research directions and ethical considerations regarding emotional vulnerability and insufficient research capacity in medical postgraduates

The predicament of emotional vulnerability and hindered research capacity development among medical postgraduates epitomizes the structural contradictions inherent in the modernization of medical education. Existing studies on this issue are limited by traditional pathologizing perspectives. Accordingly, this section proposes prospective future research directions and standardized ethical norms, aiming to shift research paradigms toward developmental and ecological analytical frameworks and resolve the ethical conflict between technical rationality and human values in medical postgraduate training research. All proposed research priorities and ethical guidelines are forward-looking suggestions to promote disciplinary progress, rather than generalized conclusions validated by sufficient evidence.

### 6.1 Diversified expansion of future research directions

1. *Interdisciplinary deconstruction of psychological resilience mechanisms*: Future research is recommended to integrate theoretical resources from neuroscience, developmental psychology, and pedagogy to elucidate the potential neural-cognitive-environmental interaction mechanisms underlying medical postgraduates’ emotional vulnerability. Additionally, subsequent studies could explore the formation mechanisms of academic self-concept and clarify the non-linear relationship between personality traits and research self-efficacy, so as to provide theoretical foundations for the development of targeted intervention tools in the future.
2. *Dynamic modeling of the educational ecosystem*: It is suggested that follow-up research construct a systematic ecosystem model for medical postgraduate training, focusing on analyzing the competitive and cooperative dynamics between academic evaluation systems and supportive resource allocation. Further exploration is needed to clarify how supervisory styles, institutional resource allocation, and academic cultural expectations jointly shape postgraduates’ research capacity development across micro and meso system levels. Specifically, qualitative and empirical studies are required to verify the functional pathways of interdisciplinary supervisory panels and flexible academic adjustment mechanisms.
3. *Evaluation of the dual-edged effects of technological tools*: The widespread application of AI-assisted research tools may bring potential risks of “technology-dependent capability degradation”. Future research should critically investigate the dual impacts of technological tools on research thinking: exploring the positive value of AI in reducing information retrieval costs and freeing cognitive resources, while systematically assessing its potential adverse effects, such as the attenuation of metacognitive abilities and weakening of core research competencies including hypothesis generation and critical thinking.

### 6.2 Core dimensions of ethical considerations

1. *Dynamic reconstruction of informed consent*: The traditional one-time informed consent model is inadequate for meeting the ethical requirements of longitudinal studies. A “procedural informed consent” mechanism needs to be established. For instance, in psychological intervention research, participants should be allowed to re-evaluate their willingness to participate based on stage-based experience, with digital platforms facilitating ongoing communication and permission management.
2. *Refined privacy protection and data governance*: The collection of emotional state and research behavior data involves high sensitivity. Research designs must adhere to the principle of “privacy by design”, employing data minimization strategies. For example, psychological assessment data should undergo graded de-identification: raw data accessible only to clinical psychology experts, while aggregated analytical results are made available to educational researchers. Simultaneously, vigilance against algorithmic bias is necessary to prevent automated decision-making based on emotional data from reinforcing stigmatizing perceptions of graduate students.
3. *Transparent management of conflicts of interest*: Role conflicts may arise when supervisors simultaneously act as researchers and intervention designers. Independent ethical review mechanisms are needed, mandating disclosure of research funding sources and any interests related to the intervention. Referencing the Declaration of Helsinki’s guidelines on researchers’ dual roles, the establishment of third-party supervisory committees can ensure that intervention research is not co-opted into a tool for performance enhancement (46).
4. *Special obligations to protect vulnerable groups*: Medical postgraduates, being both researchers and potential research subjects, possess a dual identity that readily leads to power imbalances. Ethical review must pay particular attention to: (a) Protection of autonomy: Avoiding the implicit conditioning of research participation on academic assessment; (b) Risk compensation mechanisms: Providing anonymized psychological counseling channels for postgraduates who disclose psychological distress; (c) Cultural sensitivity: Respecting differences in value orientations among postgraduates from various disciplines, preventing a one-size-fits-all standardization of intervention protocols.

### 6.3 Pathways for integrating research paradigms

1. *Research design with embedded ethics*: Employing an “ethical impact assessment” framework, systematically anticipate potential psychological risks and social controversies that research may engender during the project initiation phase. For example, when exploring the application of AI-based emotion recognition technology in graduate student stress monitoring, the potential for exacerbating self-objectification tendencies must be pre-assessed, and algorithm transparency safeguards designed accordingly.
2. *Localized adaptation of cross-cultural ethics*: When drawing on Western theories of psychological resilience, it is necessary to deconstruct their individualistic biases and construct emotional support models congruent with Eastern collectivist cultures. For instance, integrating the Confucian concept of “self-cultivation” with mindfulness training to develop intervention programs emphasizing shared responsibility within the academic community.
3. *Ethical consciousness in action research*: Encouraging the use of Participatory Action Research (PAR) methods to transform medical postgraduates from research subjects into research collaborators (47). By establishing “student ethics committees” that involve them in revising research protocols and risk assessments, both the ecological validity of the research and the ethical reflective capacity of the academic subjects can be enhanced. This facilitates a paradigm shift in training from “efficient research producers” to “integrated academic subjects,” enabling medical postgraduates to safeguard the dignity of life and spiritual fulfillment while pursuing scientific truth.

## 7 Conclusion and outlook

In summary, drawing on resilience theory and educational ecology, this study dissects the complex association between emotional vulnerability and diminished research capacity among medical postgraduates based on existing empirical literature. Theoretical analysis in this paper proposes that their linkage cannot be reduced to simple linear causality. Instead, it forms a dynamic network jointly shaped by individual psychological characteristics, educational ecosystems, and sociocultural expectations. On the basis of this theoretical deduction, we further propose that the development of medical postgraduates’ research capacity requires an “ecological cultivation” approach. At the individual level, metacognitive training can help interrupt the cognitive loop linking perfectionism and self-denial. At the supervisor level, a failure-tolerant mentoring model may reframe research setbacks as opportunities to foster academic resilience. At the institutional level, an integrated psychological-academic support system should be established. Systematic institutional innovation can mitigate conflicts between instrumental rationality and individual subjective needs. All the above-mentioned mechanistic explanations and cultivation strategies are theoretical inferences derived from the current multidisciplinary analysis, rather than empirically validated conclusions for medical postgraduate cohorts.

The core objective of this multi-tiered intervention system is to expand psychological resilience beyond individual traits and embed it within the educational ecology. This shift restores the development of research capacity to its foundational goal of holistic education. Fundamentally, the training dilemmas faced by medical postgraduates reflect broader modern contradictions manifesting within graduate education. Scholars must move beyond the binary “vulnerability–capacity” framework. Co-evolution of individual psychological resilience and educational ecosystems can reconstruct the integrity of academic subjects, enabling the cultivation of new-generation medical professionals equipped with innovative research competence and humanistic awareness. This direction represents an urgent pathway for higher education reform. It also facilitates value reconstruction that steers medical education back to its fundamental missions: “caring for life” and “shouldering the trust of patients whose lives depend on healthcare.”

### 7.1 Limitations of the study

Despite the theoretical contributions and practical implications of this study, several notable limitations should be acknowledged to guide future research and avoid overgeneralization of the findings. First, all core conclusions in this manuscript are derived from theoretical hypothetical models established via narrative synthesis of existing published literature. Notably, these theoretical inferences have not been empirically validated with targeted samples of medical postgraduates, which means the causal and correlational relationships proposed in this study require quantitative or qualitative empirical verification in the specific research context of medical graduate education.

Second, most mechanistic inferences underlying the association between emotional vulnerability and research capacity in this study are extrapolated from empirical evidence and theoretical findings based on non-medical graduate student samples. There are substantial differences in training pressure, research scenarios, academic assessment mechanisms, and professional development demands between medical postgraduates and students from other disciplines. Directly generalizing the research conclusions from non-medical graduate samples to medical postgraduates involves a considerable inferential leap, and the adaptability and applicability of the proposed mechanistic framework for medical postgraduates remain to be systematically verified.

Third, consistent with the study’s core perspective, the real-world association between emotional vulnerability and research capacity among medical postgraduates does not constitute a simple linear causal relationship. The bidirectional negative loop revealed in this study only reflects a dominant theoretical correlation trend. In practice, the two core variables interact with multiple latent variables, including individual psychological traits, supervisor mentoring styles, institutional assessment policies, and academic stress, forming complex, dynamic, and nonlinear relational mechanisms. This study fails to quantitatively delineate the nonlinear interaction effects, threshold characteristics, and contextual regulatory factors among relevant variables, and the intricate synergistic or inhibitory relationships between multi-dimensional variables still require in-depth exploration in future empirical studies.

### 7.2 Future outlook

On the basis of the above limitations, future research can be expanded in multiple dimensions. First, large-sample empirical investigations targeting medical postgraduates should be conducted to verify and revise the theoretical model proposed in this study, so as to improve the validity and targeting of the research conclusions. Second, comparative studies between medical and non-medical graduate samples can be carried out to clarify the disciplinary specificity of the linkage between emotional vulnerability and research capacity, and further refine the disciplinary adaptation boundary of relevant theoretical mechanisms. Third, quantitative empirical methods such as nonlinear regression and threshold effect analysis can be adopted to unpack the complex nonlinear relationships and dynamic evolution rules among relevant variables, thereby forming a more comprehensive and in-depth understanding of the interactive mechanism between emotional vulnerability and research capacity of medical postgraduates. Furthermore, longitudinal tracking research is expected to reveal the dynamic causal trajectory of the bidirectional negative loop over time, providing more rigorous empirical support for the optimization of medical postgraduate education and training strategies.

## Data Availability

The original contributions presented in the study are included in the article/supplementary material, further inquiries can be directed to the corresponding author.
